# The Inhibition on *MDFIC* and *PI3K/AKT* Pathway Caused by miR-146b-3p Triggers Suppression of Myoblast Proliferation and Differentiation and Promotion of Apoptosis

**DOI:** 10.3390/cells8070656

**Published:** 2019-06-29

**Authors:** Weiling Huang, Lijin Guo, Minxing Zhao, Dexiang Zhang, Haiping Xu, Qinghua Nie

**Affiliations:** 1 Department of Animal Genetics, Breeding and Reproduction, College of Animal Science, South China Agricultural University, Guangzhou 510642, China; 2 Guangdong Provincial Key Lab of Agro-Animal Genomics and Molecular Breeding and Key Lab of Chicken Genetics, Breeding and Reproduction, Ministry of Agriculture, Guangzhou 510642, China

**Keywords:** miR-146b-3p, *AKT1*, myoblast, proliferation, differentiation, apoptosis

## Abstract

Accumulating studies report that microRNAs (miRNAs) are actively involved in skeletal myogenesis. Previously, our study revealed that miR-146b-3p was related to the growth of skeletal muscle. Here, we further report that miR-146b-3p is essential for the proliferation, differentiation, and apoptosis of chicken myoblast. Elevated expression of miR-146b-3p can dramatically suppress proliferation and differentiation, and facilitate apoptosis of chicken myoblast. Besides, we identified two target genes of miR-146b-3p, *AKT1* and *MDFIC*, and found that miR-146b-3p can inhibit the *PI3K/AKT* pathway. Our study also showed that both *AKT1* and *MDFIC* can promote the proliferation and differentiation while inhibit the apoptosis of myoblast in chicken. Overall, our results demonstrate that miR-146b-3p, directly suppressing *PI3K/AKT* pathway and *MDFIC*, acts in the proliferation, differentiation, and apoptosis of myoblast in chicken.

## 1. Introduction

Skeletal muscle formation is a vital life process which has always attracted increasing attention from researchers. The development of skeletal muscle goes through the process of myoblast proliferation and differentiation into myotubes [1]. Once the course of myoblast differentiation initiates, the proliferating myoblasts are arrested from the cell cycle, ultimately resulting in the formation of myotubes [2]. In addition to the proliferation and differentiation, apoptosis of myoblast is also recognized as an essential process in skeletal muscle development. Apoptosis is a form of programmed cell death, which is a tightly regulated process [3]. Inhibition of apoptosis can lead to blockage and abnormality in skeletal muscle development, and even worse, cause inflammation and oncogenesis [4,5].

As an intricate physiological process, skeletal muscle development is regulated by a variety of transcription factors, miRNAs are included [6,7]. MiRNAs are a class of endogenous noncoding small RNAs of approximately 18 to 24 nucleotides, usually complementary to the sites in the 3′ untranslated region (3′UTR) of the target messenger RNAs (mRNAs) to repress gene expression post-transcriptionally [8,9]. Emerging studies have revealed that miRNAs are involved in the regulation of multiple life processes such as cell proliferation, cell differentiation, embryonic development, tissue inflammation, and so on [10,11,12,13]. In the process of skeletal muscle formation, the role of miRNAs cannot be ignored. So far, a host of miRNAs, particularly miR-1, miR-206, and miR-133, have been identified to be capable of mediating the skeletal muscle development [14,15,16,17,18,19]. For instance, miR-1 and miR-133 played important roles in regulating skeletal muscle proliferation and differentiation in cultured myoblast [14]. MiR-16-5p was able to repress myoblast proliferation and differentiation, and promote myoblast apoptosis [20]. MiR-133a-5p, miR-29b-1-5p, miR-34b-5p, and miR-30a-3p were characterized as important miRNAs in myoblast proliferation and differentiation [21,22,23].

MiR-146b was primarily established as a regulator in inflammation and cancer [24,25,26]. To date, only a little research has been done on miR-146b-3p, not to mention the intensive study of its roles in myoblast proliferation, differentiation, and apoptosis in skeletal muscle development. In our previous RNA-seq study (accession number: GSE91060), we found miR-146b-3p was differentially expressed in the leg muscles between the E11 and E16 of Xinghua chicken [27]. Interestingly, according to our another preliminary study (accession number GSE62971), the expression of miR-146b-3p was of higher abundance in chicken with low body weight than those with high body weight in no matter White Recessive Rock or Xinghua chicken, suggesting that miR-146b-3p might be a candidate inhibitor of chicken muscle growth [28]. Therefore, in this assay, we aim to gain insight into how miR-146b-3p acts in the regulation of the skeletal muscle development.

The *AKT1* gene, also named protein kinase B α (*PKBα*), is an important member of the *AKT* family. More notably, AKT is universally acknowledged as a core factor in the PI3K (phosphatidylinositol 3-kinase)/AKT pathway that plays critical roles in various cellular activities such as cell proliferation, cell differentiation, cell apoptosis, metabolism, protein synthesis, transcription, and so on [29,30]. A study demonstrated that in *AKT* family, *AKT1* primarily promoted and sustained the myoblast differentiation [31]. Absence of *AKT1* resulted in growth retardation and apoptosis promotion [32]. Several lines of apoptotic paradigms have also highlighted the principle role of *AKT1* in maintaining cell survival and suppressing apoptosis [33,34,35]. Besides, based on our previous study, *AKT1* appears to be one of the candidate target genes of miR-146b-3p [28]. It is of interest to uncover the potential interaction between *AKT1* and miR-146b-3p in myoblast development.

The full name of *MDFIC* is MyoD family inhibitor domain containing. There is a related gene *MDFI*, also known as *I-mfa*, which was reported to play a negative role in regulating the transcription of MyoD family in the differentiation of fibroblast [36]. MDFIC contains a cysteine-rich C-terminal region that shares a high degree homology with I-mfa. The construction similarity between MDFIC and I-mfa suggests that MDFIC can be an inhibitor of myoblast differentiation. However, to our surprise, *MDFIC* tended to promote cell differentiation as well as cell proliferation in our current study. Thus, it is definitely of considerable interest to further determine how myoblast responds when *MDFIC* is silenced or over-expressed.

In this study, we investigate the function of miR-146b-3p on the proliferation, apoptosis, and differentiation of myoblast. We found that miR-146b-3p can target *AKT1* and *MDFIC*, and suppress *PI3K/AKT* pathway. In addition, we also verified the effects of *AKT1* and *MDFIC* on myoblast proliferation, apoptosis, and differentiation in chicken.

## 2. Materials and Methods

### 2.1. Ethics Statement

All animal experiments in this study were approved by the Animal Care Committee of South China Agricultural University (Guangzhou, People’s Republic of China) (approval number: SCAU#0014).

### 2.2. Cell Culture

Chicken primary myoblasts (CPMs) were isolated from the leg muscles of 11-embryo-age chicken and cultured in the Roswell Park Memorial Institute (RPMI)-1640 medium (Gibco, Grand Island, NY, USA) supplemented with 20% fetal bovine serum (Gibco, Grand Island, NY, USA) and 0.2% penicillin/streptomycin solution (Invitrogen, Carlsbad, CA, USA). The differentiation of myoblasts was induced by RPMI-1640 medium consisting of 2% horse serum (Hyclone, Logan, UT, USA) and 0.2% penicillin/streptomycin (Invitrogen, Carlsbad, CA, USA). Quail muscle clone 7 (QM-7) cells were cultured in Medium 199 basic (Gibco, Grand Island, NY, USA) with 10% fetal bovine serum (Gibco, Grand Island, NY, USA), 10% tryptose phosphate broth solution (Sigma, Louis, MO, USA), and 0.2% penicillin/streptomycin (Invitrogen, Carlsbad, CA, USA). DF-1 cell lines of chicken embryo fibroblast (DF-1 cells) were cultured in high-glucose Dulbecco’s modified Eagle’s medium (DMEM) (Gibco, Grand Island, NY, USA) with 10% fetal bovine serum (Gibco, Grand Island, NY, USA) and 0.2% penicillin/streptomycin (Invitrogen, Carlsbad, CA, USA). All cells were cultured in 5% CO_2_ at 37 °C.

### 2.3. RNA Isolation, Complementary DNA (cDNA) Synthesis, and Quantitative Real-Time PCR (q-PCR)

Total RNA from tissues or cells was extracted using Hi Pure Total RNA Mini Kit (Magen, Guangzhou, China) following the manufacturer’s protocol. The Prime ScriptTM RT reagent kit with gDNA Eraser (Perfect Real Time) (TaKaRa, Otsu, Japan) was used in cDNA synthesis for mRNA. The mRNA expression levels were detected by q-PCR with Bio-rad CFX96 instrument (Bio-Rad, Hercules, CA, USA) using iTAP^TM^ universal SYBR GREEN superMIX (Bio-Rad, Hercules, CA, USA). The reference genes were *U6* and *GAPDH* in the process of quantification of miRNA and mRNA, respectively. All primers were designed by Premier Primer 5.0 software (Premier Biosoft International, Palo Alto, CA, USA) and synthesized by TSINGKE Biotech (Guangzhou, China). The primers of the q-PCR are listed in Table 1. Relative mRNA expression levels were calculated using the 2^^−ΔΔCT^ method (ΔCT = CT_target gene_ − CT_reference gene_, ΔΔCT = ΔCT_treat group_ − ΔCT_control group_).

### 2.4. RNA Oligonucleotides and Plasmids Construction

Gga-miR-146b-3p mimic, miR-146b-3p inhibitor, mimic negative control (NC), inhibitor NC, small interfering RNA (siRNA), and siRNA negative control (si-NC) used in this study were synthesized by RiboBio (Guangzhou, China). The oligonucleotides sequences used in the study are listed in Table 2.

The complete CDS sequence of *AKT1* and *MDFIC* were respectively amplified by PCR and were cloned into the expression vector, pcDNA3.1 (Promega, Madison, WI, USA) by using the Xba I and Xho I restriction sites through pJET1.2 cloning vector. The plasmids were named pcDNA3.1-*AKT1* and pcDNA3.1-*MDFIC*, respectively. The segment sequence of the 3′UTR of *AKT1* and *MDFIC* that contained the putative gga-miR-146b-3p binding sequence were amplified, the segment sequence of the 3′UTR of *AKT1* was subcloned into Xba I and Xho I sites in the pmiR-GLO dual-luciferase while the segment sequence of the 3′UTR of *MDFIC* was subcloned into Hind III and Not I sites reporter vector (Promega, Madison, WI, USA). The 3′UTR mutant plasmids were obtained by converting the binding site of miR-146b-3p from CCATAGG to TTCGCAA. PCR amplification of the mutants and DPNI digestion removed the parental DNA. The primers used to construct the plasmids are listed in Table 3.

### 2.5. MiRNA Targets Prediction and RNA Hybrid Detection

miRDB (http://mirdb.org/miRDB/) was used to predict the target genes of gga-miR-146b-3p. RNAhybrid (http://bibiserv2.cebitec.uni-bielefeld.de/rnahybrid/) detection was used to calculate the combined minimum free energy (MFE) of gga-miR-146b-3p and the 3′UTR of *AKT1* and *MDFIC* to determine the binding stability of the duplex.

### 2.6. Cell Transfection

Transfections were performed with Lipofectamine 3000 reagent (Invitrogen, Carlsbad, CA, USA) according to the manufacturer’s protocol with at least three replications. The transfection concentration of the oligonucleotides was 50 nM. The transfection concentration of the plasmids was followed as: 0.1 μg/well for 96-well plate, 0.25 μg/well for 24-well plate, 1 μg/well for 12-well plate, 2.5 μg/well for 6-well plate.

### 2.7. Dual-Luciferase Reporter Assay

DF-1 cells were seeded in 96-well plates and co-transfected with plasmid of wild-type 3′UTR or mutant 3′UTR with mimic or mimic-NC. After 48 h, the luciferase activity was detected using a Dual-GLO Luciferase Assay System Kit (Promega, Madison, WI, USA) following its instruction. The firefly luciferase and Renilla luminescence activities were detected using multi-function microplate reader (Biotek, Winooski, VT, USA).

### 2.8. Immunofluorescence

Cells seeded in 12-well plates were fixed in 4% formaldehyde for 20 min and washed three times with PBS for 5 min after 48-h transfection. Subsequently, the cells were treated with 0.1% Triton X-100 for 15 min and blocked with goat serum for 30 min and then incubated with MyHC antibody (B103; DHSB, USA; 0.5 μg/mL) overnight at 4 °C. After that Fluorescein (FITC)-conjugated AffiniPure Goat AntiMouse IgG (H + L) (BS50950; Bioworld, Minneapolis, MN, USA; 1:50) was added to the cells and the cells were incubated at room temperature for 1 h. Besides, the cell nuclei were stained with DAPI for 5 min. And lastly, Leica DMi8 fluorescent microscope (Leica, Wetzlar, Germany) was used to obtain the images, and ImageJ software was used to measure the percentage of the total image area covered by the myotubes.

### 2.9. 5-Ethynyl-2′-deoxyuridine (EdU) Assay

EdU Assay was performed to test cell proliferation using Cell-Light EdU DNA Cell proliferation Kit (RiboBio, Guangzhou, China). CPMs and QM-7 cells were cultured in 96-well plates for transfection. Briefly, after 48-h transfection, the cells were incubated with 50 nM EdU for 2 h at 37 °C; the cells were then fixed with 4% paraformaldehyde and stained with Apollo dye solution for proliferating cells. Nucleic acids in all cells were stained with Hoechst 33342. Leica DMi8 fluorescent microscope was used to capture five randomly selected fields to visualize the EdU-stained cells. The proliferation rate was the ratio of the number of EdU-stained cells to the number of Hoechst 33342-stained cells.

### 2.10. Flow Cytometric Analysis of Cell Cycle

CPMs or QM-7 cells were cultured in 12-well plates. After transfection for 48 h, cells were collected and then fixed in 75% ethanol overnight at −20 °C. Subsequently, the fixed cells were stained with propidium iodide (Sigma, Louis, MO, USA) (50μg/mL) containing 10μg/mL RNase A (TaKaRa, Otsu, Japan) and 0.2% (v/v) Triton X-100 (Sigma, Louis, MO, USA), and then incubated for 30 min at 37 °C in the dark. Flow cytometric analysis was performed on a flow cytometer (Beckman, Miami, FL, USA) and data was processed using FlowJo7.6 software.

### 2.11. Flow Cytometric Analysis of Cell Apoptosis

CPMs or QM-7 cells were seeded in 12-well plates and after transfection for 48 h the cells were collected. Then the cells were stained through an Annexin V-FITC apoptosis detection kit (Beyotime, Shanghai, China) and analyzed by a flow cytometer (Beckman, Miami, FL, USA), following the manufacturers protocol.

### 2.12. Western Blot Assay

Cellular proteins were extracted using ice-cold radio immunoprecipitation (RIPA) lysis buffer (Beyotime, Shanghai, China) with 1 mM phenylmethane sulfonyl fluoride (PMSF) protease inhibitor (Beyotime, Shanghai, China). The protein samples were separated by 10% SDS-PAGE at a voltage of 100 volts for 20 minutes and then at 120 volts for 60 minutes. Subsequently, the proteins were transferred onto a nitrocellulose membrane (Whatman, Maidstone, UK) or a polyvinylidene fluoride (PVDF) membrane (Bio-Rad, Hercules, CA, USA), and then probed using antibodies according to standard procedures. The antibodies used for Western blots and their dilutions are as follows: Cleaved Caspase-8 (Asp391) (18C8) Rabbit mAb (Cell Signaling Technology, Boston, MA, USA; 1:1000), Anti-Caspase-9 antibody [E23] (Abcam, London, UK; 1:1000), mouse anti-MyHC antibody (Bioss, Beijing, China; 1:1000), rabbit anti-Lamin B antibody (Bioss, Beijing, China; 1:500), rabbit anti-AKT1 (Bioss, Beijing, China; 1:500), rabbit anti-phospho-AKT1 (Bioss, Beijing, China; 1:500), rabbit Anti-phospho-AKT antibody (Bioss, Beijing, China; 1:300), and mouse anti-GAPDH (Boster, Wuhan, China; 1:2000). Finally, the secondary antibody (Boster, Wuhan, China) containing horseradish peroxidase chain reaction (HRP) anti-rabbit/mouse IgG antibody (Boster, Wuhan, China) was incubated in a 1:10,000 dilution.

### 2.13. Statistical Analysis

All data are derived from at least three replicates of experimental processing. Statistically significant differences were calculated using Student’s *t*-test. Results are shown as mean ± S.E.M. (standard error of the mean) and the difference was considered as statistically significant when the *p*-value < 0.05 (*) or *p*-value < 0.01 (**).

## 3. Results

### 3.1. miR-146b-3p Contributes to Cell Cycle Arrest in Myoblast and Suppresses Cell Proliferation

miR-146b-3p was successfully overexpressed with the mimic and silenced with the inhibitor respectively in CPMs and QM-7 cells, indicating that the mimic and inhibitor of miR-146b-3p were available for the following verification experiments (Supplementary Figure S1a–d). We first investigated the effects of miR-146b-3p on myoblast proliferation. Flow cytometry, q-PCR, and EdU assays were performed in CPMs and QM-7 cells, respectively.

**Figure 1:**
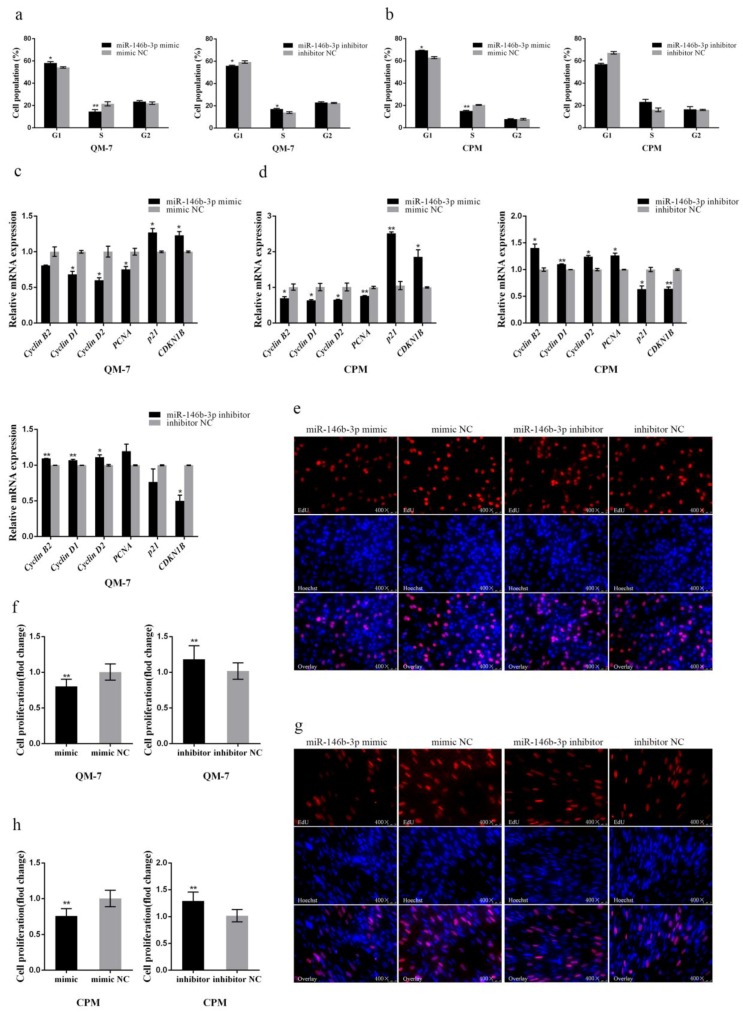
gga-miR-146b-3p inhibits myoblast proliferation. (**a**,**b**) Cell cycle analysis of QM-7 cells and chicken primary myoblasts (CPMs) after transfecting miR-146-3p mimic or inhibitor. (**c**,**d**) Relative mRNA expression of the cell cycle-related genes after transfection of miR-146-3p mimic or inhibitor in QM-7 cells and CPMs. (**e**) 5-Ethynyl-2′-deoxyuridine (EdU) staining of QM-7 cells after transfection of miR-146-3p mimic or inhibitor. (**f**) The fold change of proliferation rates of QM-7 cells with miR-146-3p mimic or inhibitor. (**g**) EdU staining of CPMs after transfection of miR-146-3p mimic or inhibitor. (**h**) The fold change of proliferation rates of CPMs with miR-146-3p mimic or inhibitor. Results of all groups are shown as mean ± standard error of the mean (S.E.M.) of three independent assessment methods. Statistical significance of the mean difference was assessed using unpaired two-sample *t*-tests. * *p* < 0.05; ** *p* < 0.01. NC, negative control.

After the 48-h transfection in CPMs and QM-7 cells with miR-146b-3p mimic and inhibitor, flow cytometry analysis of the cell cycle was performed. The results showed that overexpression of miR-146b-3p can arrest QM-7 cells and CPMs in the G1 phase, causing the increase of the cell population in the G1 phase while distinct decrease in the S phase (Figure 1a,b). Moreover, the inhibition of miR-146b-3p revealed opposite results (Figure 1a,b). Overall, miR-146b-3p can block the cell cycle progress, thereby inhibiting the myoblast proliferation.

In addition, we tested the mRNA expression levels of several cell cycle-related genes in CPMs and QM-7 cells by q-PCR. The results showed that miR-146b-3p overexpression can reduce the mRNA expression of cell cycle-promoting genes, such as *Cyclin B2*, *Cyclin D1*, *Cyclin D2*, and *PCNA*, while increase cell cycle-inhibiting genes like *p21* and *CDKN1B* (Figure 1c,d). The inhibition of miR-146b-3p revealed opposite results to the miR-146b-3p mimic groups (Figure 1c,d). Thus, miR-146b-3p can modulate the cell cycle-related genes to inhibit myoblast proliferation.

To further determine the effects of miR-146b-3p on myoblast proliferation, we also performed EdU staining experiment to observe the cell proliferation status after transfecting miR-146b-3p mimic or inhibitor into CPMs and QM-7 cells respectively. The results showed whether in CPMs or QM-7 cells, the ratio of proliferative cells of the miR-146b-3p mimic group was significantly reduced compared with that of the control group (Figure 1e–h). Conversely, after the inhibition of miR-146b-3p, the ratio of cells in proliferation phase was extremely increased (Figure 1e–h). These results indicate that miR-146b-3p can inhibit the proliferation of myoblast.

### 3.2. miR-146b-3p Inhibits Myoblast Differentiation

**Figure 2:**
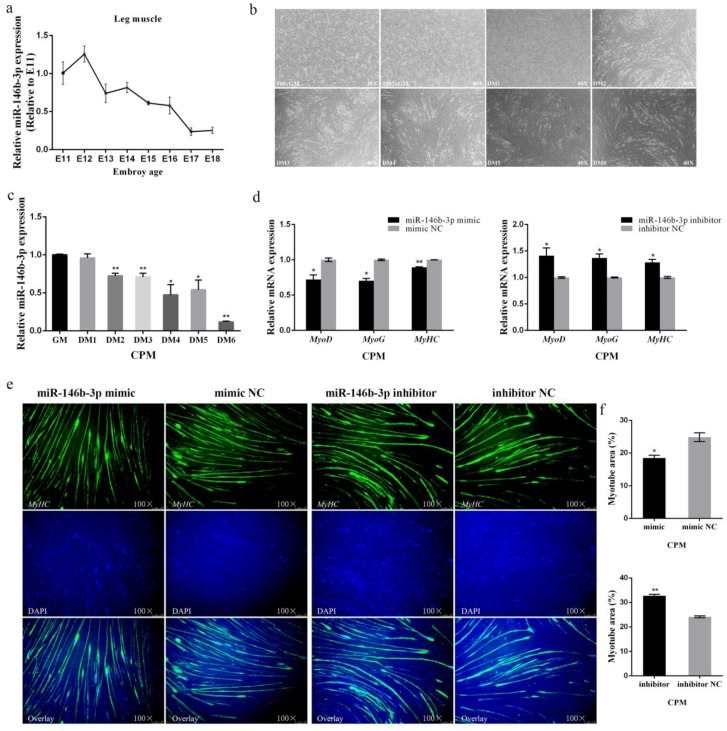
miR-146b-3p suppresses myoblast differentiation. (**a**) Relative expression of miR-146b-3p in Xinghua chicken leg muscle from E11 to E18. (**b**) Morphology of CPMs cultured in growth medium (GM) and differentiation medium (DM) from day 1 to 6. (**c**) The relative mRNA expression of miR-146b-3p during CPMs-induced differentiation. (**d**) Relative mRNA expression of the cell differentiation-related genes after transfection of miR-146-3p mimic or inhibitor in CPMs. (**e**) Immunofluorescence of MyHC after transfection of miR-146-3p mimic or inhibitor in CPMs. (**f**) Comparison of the area of myotubes described in (**e**). Results of all groups are shown as mean ± S.E.M. of three independent assessment methods. Statistical significance of the mean difference was assessed using unpaired two-sample *t*-tests. * *p* < 0.05; ** *p* < 0.01. NC, negative control.

To observe the expression of miR-146b-3p in leg muscle during chicken embryogenesis, we constructed an expression profile of miR-146b-3p from E11 to E18, which showed that the expression of miR-146b-3p declined in fluctuation from E12 to E18 (Figure 2a). Besides, we also induced CPMs to differentiate in vitro (Figure 2b) and found the expression of miR-146b-3p declined when the cells underwent differentiation (Figure 2c). These results indicate the potential inhibitory effects of miR-146b-3p on myoblast differentiation.

**Figure 3:**
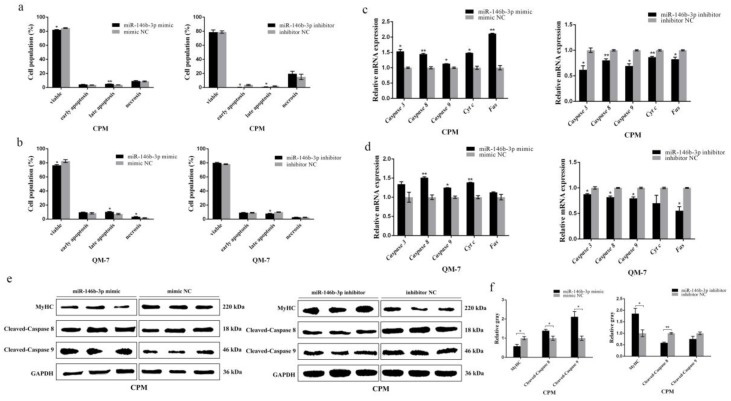
miR-146b-3p promotes myoblast apoptosis. (**a**) Flow cytometry of Annexin V-FITC and propidium iodide (PI) dual staining detecting the apoptosis of CPMs after transfection of miR-146-3p mimic or inhibitor. (**b**) Flow cytometry of Annexin V-FITC and propidium iodide (PI) dual staining detecting the apoptosis of QM-7 cells after transfection of miR-146-3p mimic or inhibitor. (**c**) Relative mRNA expression of the cell apoptosis-related genes after transfection of miR-146-3p mimic or inhibitor in CPMs. (**d**) Relative mRNA expression of the cell apoptosis-related genes after transfection of miR-146-3p mimic or inhibitor in QM-7 cells. (**e**) The protein levels of MyHC, cleaved-caspase 8, and cleaved-caspase 9 after the transfection of mimic or inhibitor in CPMs. (**f**) Gray value analysis of protein bands in (**e**). Results of all groups are shown as mean ± S.E.M. of three independent assessment methods. Statistical significance of the mean difference was assessed using unpaired two-sample *t*-tests. * *p* < 0.05; ** *p* < 0.01. NC, negative control.

Therefore, we moved on to determine the role of miR-146b-3p in myoblast differentiation. It was found that overexpression of miR-146b-3p remarkably decreased the mRNA expression of myoblast differentiation-related genes including *MyoD*, *MyoG*, and *MyHC*, whereas, inhibition of miR-146b-3p increased the mRNA expression of *MyoD*, *MyoG*, and *MyHC* (Figure 2d and Supplementary Figure S2). In addition, the protein expression of MyHC was decreased after the overexpression of miR-146b-3p in CPMs, while the protein expression of MyHC was enriched after the inhibition of miR-146b-3p as exhibited (Figure 3e,f).

Immunofluorescence was also performed to track the effects of miR-146b-3p on myoblast differentiation. The results showed that overexpression of miR-146b-3p blocked the formation of myotubes, whereas, inhibition of miR-146b-3p contributed to the formation of myotubes (Figure 2e,f). 

All the results above potently state that miR-146b-3p is capable of inhibiting the myoblast differentiation.

### 3.3. miR-146b-3p Promotes Myoblast Apoptosis

Apart from proliferation and differentiation of myoblast, the apoptosis of myoblast is also of great significance in the development of skeletal muscle. Therefore, we also paid attention to the potential effects of miR-146b-3p on myoblast apoptosis. 

Flow cytometry analysis performed to detect the myoblast apoptosis showed that the expression of miR-146b-3p promoted myoblast apoptosis (Figure 3a,b). The mRNA expression of some well-known apoptosis-related genes, including *Caspase 3*, *Caspase 8*, *Caspase 9*, *Cyt c*, and *Fas*, was detected after the overexpression and inhibition of miR-146b-3p in CPMs and QM-7 cells, respectively. The results showed that apoptosis-related genes were all upregulated after the overexpression of miR-146b-3p, while the apoptosis-related genes were downregulated after the inhibition of miR-146b-3p (Figure 3c,d). Furthermore, the protein expression of cleaved-caspase 8 and cleaved-caspase 9 was detected, and the results showed that miR-146b-3p was able to upregulate the protein expression of cleaved-caspase 8 and cleaved-caspase 9 (Figure 3e,f). Moreover, the expression of miR-146b-3p suppressed the protein level of lamin B (Supplementary Figure S3a,b). These results indicate miR-146b-3p has a positive regulatory effect on myoblast apoptosis.

### 3.4. miR-146b-3p Targets AKT1 and MDFIC and Downregulates PI3K/AKT Pathway Activity

**Figure 4:**
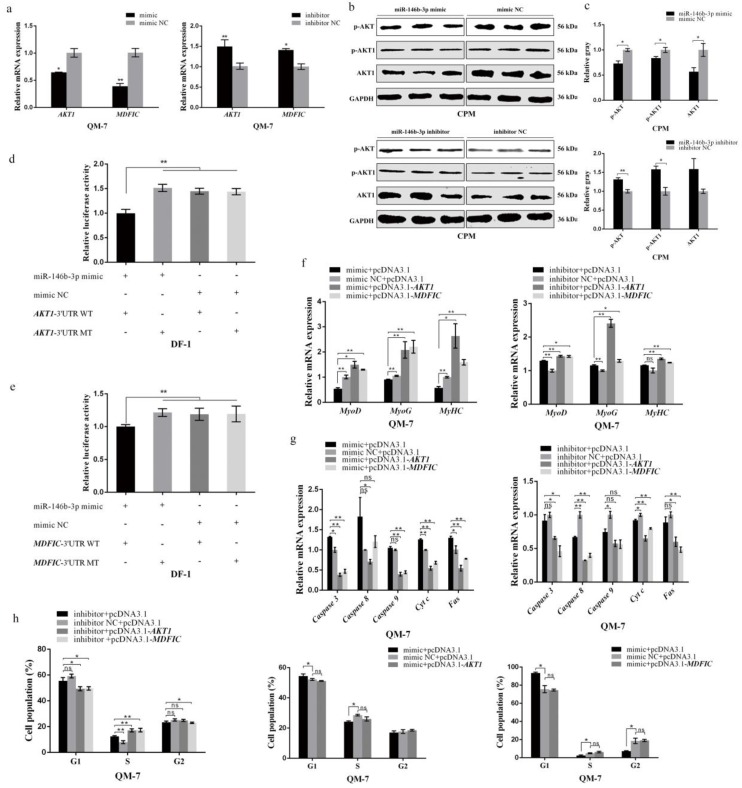
miR-146b-3p targets *AKT1* and *MDFIC* and downregulates *PI3K/AKT* pathway activity. (**a**) Relative mRNA expression of *AKT1* and *MDFIC* in QM-7 cells after overexpression or inhibition of miR-146b-3p. (**b**) The phosphorylation levels of AKT and AKT1 and the protein expression of AKT1 after transfecting miR-146b-3p mimic or inhibitor in CPMs. (**c**) Gray value analysis of protein bands in (b). (**d**) Dual-luciferase report assay performed after co-transfecting the wild type or mutant 3′UTR of *AKT1* with miR-146b-3p mimic or mimic NC in DF-1 cells. (**e**) Dual-luciferase report assay performed after co-transfecting the wild type or mutant 3′UTR of *MDFIC* with miR-146b-3p mimic or mimic NC in DF-1 cells. (**f**) Relative mRNA expression of the cell differentiation-related genes after co-transfection. (**g**) Relative mRNA expression of the cell apoptosis-related genes after co-transfection. (**h**) Cell cycle analysis of QM-7 cells after co-transfection. Results of all groups are shown as mean ± S.E.M. of three independent assessment methods. Statistical significance of the mean difference was assessed using unpaired two-sample *t*-tests. * *p* < 0.05; ** *p* < 0.01. NC, negative control.

It is well established that miRNAs function mainly by targeting their target genes. To further explore how miR-146b-3p works in the regulation of skeletal muscle development, we tried to predict its possible target genes on the miRDB website. We found that miR-146b-3p was predicted to be able to target *AKT1* and *MDFIC*. The seed sequences of miR-146b-3p were perfectly complementary to the 3′UTR region of *AKT1* and *MDFIC* (Supplementary Figure S4a). Besides, the MFE between miR-146b-3p and *AKT1* 3′UTR was approximately −21.9 kcal/mol (Supplementary Figure S4b) and *MDFIC* 3′UTR was −24.2 kcal/mol (Supplementary Figure S4c), indicating they possess a stable combination. The mRNA expression of *AKT1* and *MDFIC* can be suppressed after overexpression of miR-146b-3p while enhanced after inhibition of miR-146b-3p (Figure 4a). As a core factor in *PI3K/AKT* pathway, the phosphorylation of AKT can affect *PI3K/AKT* pathway activity. Subsequently, we tested the expression and phosphorylation of AKT1 protein and phosphorylation of AKT, it was found that miR-146b-3p was able to negatively regulate *PI3K/AKT* pathway activity (Figure 4b,c).

Moreover, the dual-luciferase report experiments were performed and showed that the matching of miR-146b-3p and the 3′UTR region of *AKT1* or *MDFIC* led to a significant suppression of luciferase activity, whereas mutation of target sites failed to weaken the luciferase activity (Figure 4d,e), coinciding with the mRNA expression analysis results above.

Finally, we conducted a series of recovery validation experiments on the function of myoblast proliferation, differentiation, and apoptosis. The effects of miR-146b-3p on myoblast proliferation, differentiation, and apoptosis were all restored or even reversed by the co-expression assays (Figure 4f–h). 

These results sufficiently indicate the direct target relationships between miR-146b-3p and *AKT1* or *MDFIC*. To further learn about how miR-146b-3p works by interacting with *AKT1* and *MDFIC*, we explored the effects of *AKT1* and *MDFIC* on myoblast proliferation, differentiation, and apoptosis.

### 3.5. Both AKT1 and MDFIC Can Facilitate the Proliferation of Myoblast

To determine how *AKT1* and *MDFIC* exert biological effects on myoblast proliferation, *AKT1* and *MDFIC* were successfully overexpressed and silenced in CPMs and QM-7 cells, respectively (Supplementary Figure S1e–l). The flow cytometry, q-PCR, and EdU assays were then performed. 

**Figure 5:**
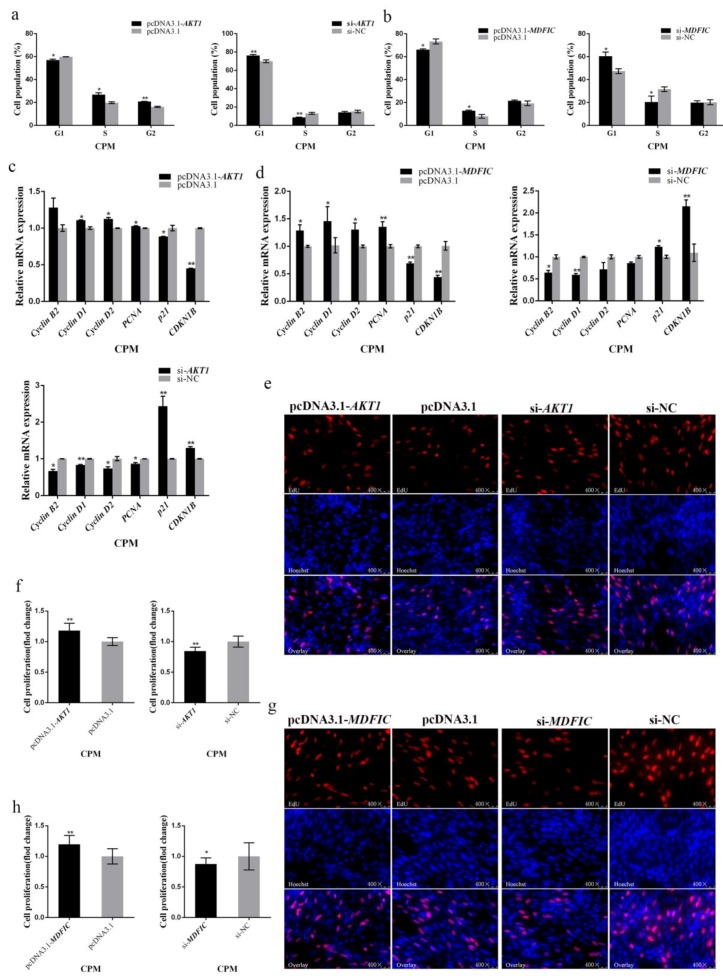
*AKT1* and *MDFIC* promote myoblast proliferation. (**a**) Cell cycle analysis of CPMs after overexpression or inhibition of *AKT1*. (**b**) Cell cycle analysis of CPMs after overexpression or inhibition of *MDFIC*. (**c**) Relative mRNA expression of the cell cycle-related genes after overexpression or inhibition of *AKT1* in CPMs. (**d**) Relative mRNA expression of the cell cycle-related genes after overexpression or inhibition of *MDFIC* in CPMs. (**e**) EdU staining of CPMs after overexpression or inhibition of *AKT1*. (**f**) The fold change of proliferation rates of CPMs with pcDNA3.1-*AKT1* or si-*AKT1*. (**g**) EdU staining of CPMs after overexpression or inhibition of *MDFIC*. (**h**) The fold change of proliferation rates of CPMs with pcDNA3.1-*MDFIC* or si-*MDFIC*. Results of all groups are shown as mean ± S.E.M. of three independent assessment methods. Statistical significance of the mean difference was assessed using unpaired two-sample *t*-tests. * *p* < 0.05; ** *p* < 0.01. NC, negative control.

Flow cytometry analysis of the cell cycle revealed that in both CPMs and QM-7 cells, overexpression of *AKT1* or *MDFIC* significantly promoted cell transition from G1 phase to S phase, while knockdown of *AKT1* or *MDFIC* significantly blocked cell transition from G1 phase to S phase (Figure 5a,b and Supplementary Figure S5a,b). These results illustrate that both *AKT1* and *MDFIC* contribute to the cell cycle progress.

We also detected the mRNA expression levels of some cell cycle-related genes after overexpressing or silencing *AKT1* or *MDFIC* in CPMs and QM-7 cells by q-PCR, respectively. The results showed that both *AKT1* and *MDFIC* overexpression can upregulate the cell cycle promoting genes including *Cyclin B2*, *Cyclin D1*, *Cyclin D2*, and *PCNA*, while downregulate cell cycle inhibiting genes like *p21* and *CDKN1B* (Figure 5c,d and Supplementary Figure S5c,d). It turned out to be an opposite result after silencing *AKT1* or *MDFIC* (Figure 5c,d and Supplementary Figure S5c,d). These results indicate that both *AKT1* and *MDFIC* can regulate the cell cycle-related genes and participate in promoting myoblast proliferation. 

In addition, the EdU assay showed that in both CPMs and QM-7 cells, the proportion of proliferative cells remarkably increased, whereas the knockdown of *AKT1* decreased the proportion of proliferative cells (Figure 5e,f and Supplementary Figure S5e,f); and, *MDFIC* showed the same effect (Figure 5g,h and Supplementary Figure S5g,h).

Taken together, we draw a conclusion that both *AKT1* and *MDFIC* can promote myoblast proliferation in chicken.

### 3.6. In Terms of Myoblast Differentiation, Both AKT1 and MDFIC Exhibit Positive Impacts

**Figure 6:**
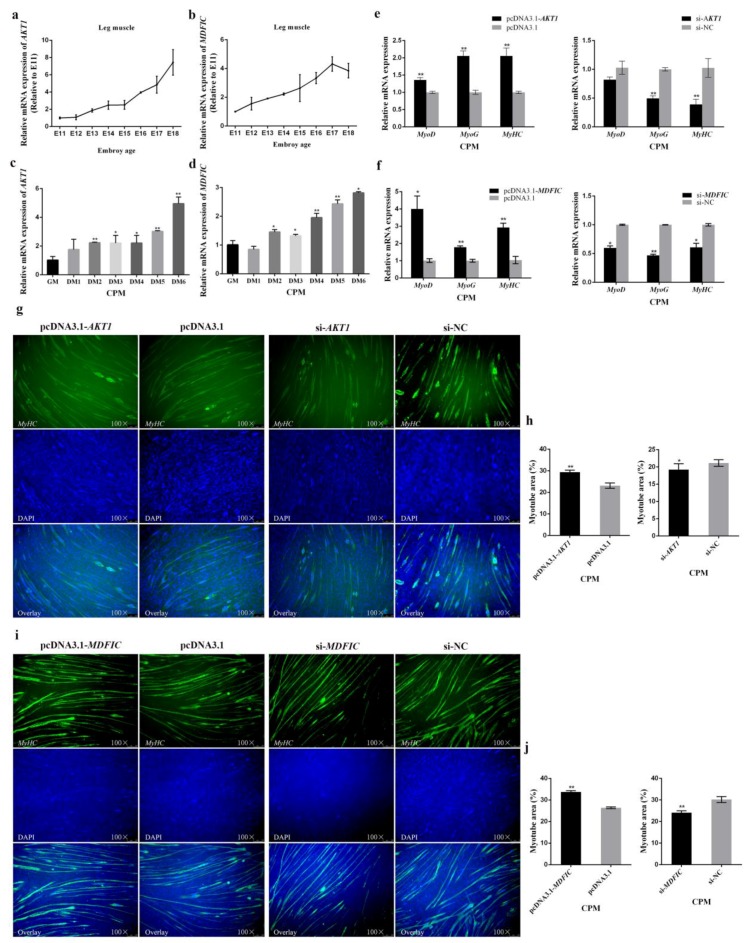
Both *AKT1* and *MDFIC* promote myoblast differentiation, while suppress myoblast apoptosis. (**a**) Relative expression of *AKT1* in Xinghua chicken leg muscle from E11 to E18. (**b**) Relative expression of *MDFIC* in Xinghua chicken leg muscle from E11 to E18. (**c**) Relative mRNA expression of *AKT1* during CPMs induced differentiation. (**d**) Relative mRNA expression of *MDFIC* during CPMs-induced differentiation. (**e**,**f**) Relative mRNA expression of the cell differentiation-related genes after overexpression or inhibition of *AKT1* or *MDFIC* in CPMs. (**g**,**i**) Immunofluorescence of MyHC after overexpression or knockdown of *AKT1* and *MDFIC* in CPMs. (**h**,**j**) Comparison of the area of myotubes described in (**g**,**i**). Results of all groups are shown as mean ± S.E.M. of three independent assessment methods. Statistical significance of the mean difference was assessed using unpaired two-sample *t*-tests. * *p* < 0.05; ** *p* < 0.01. NC, negative control.

During the leg muscle development of Xinghua chicken in the embryonic period, the expression of *AKT1* and *MDFIC* was gradually upregulated from E11 to E18 (Figure 6a,b), suggesting that *AKT1* and *MDFIC* may be involved in the regulation of the differentiation of skeletal myoblast. 

**Figure 7:**
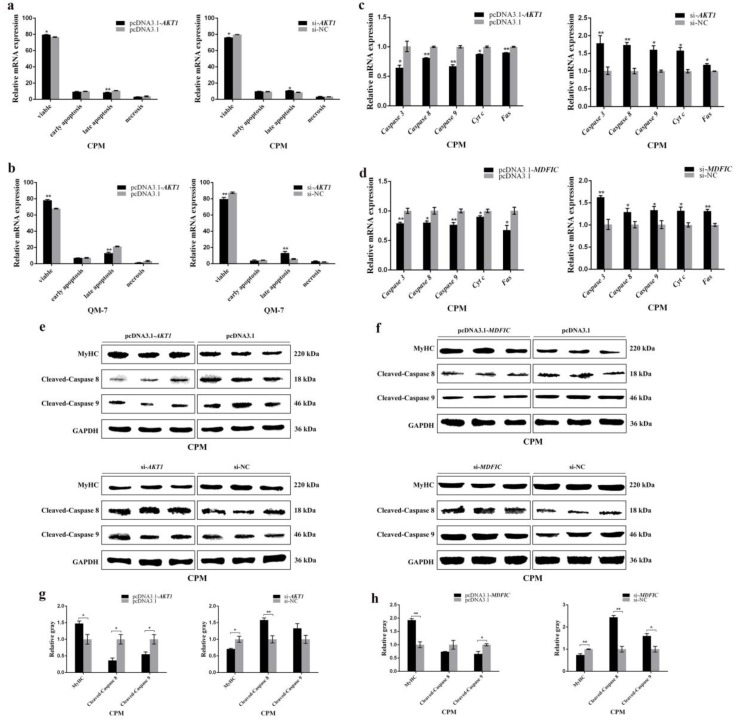
*AKT1* shows an inhibitory effect on myoblast apoptosis and so does *MDFIC*. (**a**,**b**) Flow cytometry analysis of Annexin V-FITC and PI dual staining detecting the apoptosis of CPMs after transfection of pcDNA3.1-*AKT1* or si-*AKT1*. (**c**) Relative mRNA expression of the cell apoptosis-related genes after transfection of pcDNA3.1-*AKT1* or si-*AKT1* in CPMs. (**d**) Relative mRNA expression of the cell apoptosis-related genes after transfection of pcDNA3.1-*MDFIC* or si-*MDFIC* in CPMs. (**e**,**f**) The protein levels of MyHC, cleaved-caspase 8, and cleaved-caspase 9 after the overexpression or silence of *AKT1* and *MDFIC* in CPMs. (**g**,**h**) Gray value analysis of protein bands in (**e**,**f**). Results of all groups are shown as mean ± S.E.M. of three independent assessment methods. Statistical significance of the mean difference was assessed using unpaired two-sample *t*-tests. * *p* < 0.05; ** *p* < 0.01. NC, negative control.

With the CPMs differentiating, the mRNA expression levels of *AKT1* and *MDFIC* were getting higher (Figure 6c,d). The overexpression of *AKT1* or *MDFIC* significantly upregulated the mRNA expression of the myogenic marker genes, and it showed an opposite effect after the interference of *AKT1* or *MDFIC* (Figure 6e,f and Supplementary Figure S6a,b). Moreover, the protein expression of MyHC was also upregulated after the overexpression of *AKT1* or *MDFIC* in CPMs, and it was downregulated after the knockdown of *AKT1* or *MDFIC* in CPMs (Figure 7e,f).

Immunofluorescence staining was also performed and the results showed that overexpression of *AKT1* or *MDFIC* facilitated the formation of myotubes, while it showed opposite results after silencing *AKT1* or *MDFIC* (Figure 6g–j). 

All these results demonstrate that both *AKT1* and *MDFIC* can promote the differentiation of myoblast.

### 3.7. AKT1 Shows an Inhibitory Effect on Myoblast Apoptosis and so Does MDFIC

As described earlier, in the development of myoblast, cell apoptosis is also an important process. We wondered if miR-146b-3p can interact with *AKT1* or *MDFIC* in regulating myoblast apoptosis. 

Flow cytometry results showed that myoblast apoptosis was suppressed after the overexpression of *AKT1* in QM-7 cells and CPMs (Figure 7a,b). Besides, the mRNA expression levels of several well-known apoptosis-related genes including *Caspase 3*, *Caspase 8*, *Caspase 9*, *Cyt c*, and *Fas* were detected. The results showed that the apoptosis-related genes were downregulated after the overexpression of *AKT1* or *MDFIC*, while apoptosis-related genes were upregulated with the knockdown of *AKT1* or *MDFIC* (Figure 7c,d and Supplementary Figure S6c,d). In addition, the protein levels of cleaved-caspase 8 and cleaved-caspase 9 were downregulated by the expression of *AKT1* and *MDFIC* (Figure 7e–h). Moreover, both *AKT1* and *MDFIC* can promote the protein expression of lamin B (Supplementary Figure S3c–f). The regulatory effects of *AKT1* and *MDFIC* on apoptosis-related genes were contrasting to those of miR-146b-3p, suggesting that miR-146b-3p can promote the chicken myoblast apoptosis by inhibiting *AKT1* and *MDFIC*.

## 4. Discussion

**Figure 8:**
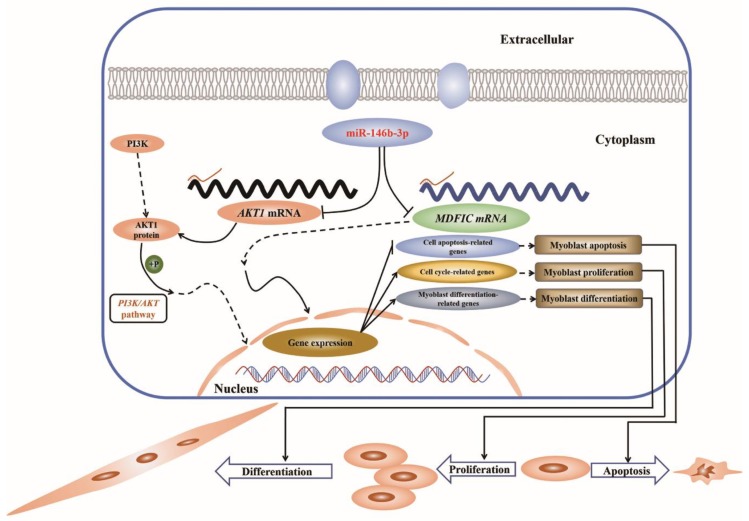
Model of miR-146b-3p mediated regulatory mechanism in myoblast proliferation, differentiation, and apoptosis. In simple terms, miR-146b-3p downregulates the expression of *AKT1* and *MDFIC* by targeting the 3′UTR of their mRNA. Both the phosphorylation of AKT1 and AKT can trigger the activation of *PI3K/AKT* pathway, thereby promoting the expression of cell cycle-related genes and myoblast differentiation-related genes and suppress cell apoptosis-related genes, which in turn facilitate cell proliferation and differentiation and inhibit apoptosis. In addition, the expression of *MDFIC* can also lead to the same effect.

In this study, we characterized the role of miR-146b-3p in the regulation of skeletal muscle development. We confirmed that miR-146b-3p can regulate myoblast proliferation, differentiation, and apoptosis via negatively regulating the activities of the *PI3K/AKT1* pathway and another target gene *MDFIC* (Figure 8).

It has been widely reported that miR-146b-3p mainly associates with glioma and thyroid tumor [26,37], while there is no research revealing its significant participation in skeletal muscle development. In our previous study, miR-146b-3p was proposed as a promising candidate gene related to the muscle development of chicken [28]. Here, we have performed a series of experiments in vitro to further determine the biological function of miR-146b-3p in skeletal muscle development. In verification, both mimic and inhibitor can cause large biological changes, some changes may be small, but they also have statistically significant differences. For example, compared with the control, inhibiting miR-146b-3p resulted in a transition of the cell cycle from G1 to S and the change of cell percentage in S is not particularly large. But the values in the group are so similar, resulting in a small S.E.M. in the group and a significant difference between the groups. Experiments such as EdU assay, Immunofluorescence assay, and the caspase cleavage assay have directly shown that these differences can cause large biological changes in myoblast proliferation, differentiation, and apoptosis. In fact, the expression change of one gene is often accompanied by the expression changes of multiple genes, which all together can lead to a specific phenotype. For instance, miR-16 was able to simultaneously target *SENE1*, *Bcl6*, and *FOXO1*, which altogether resulted in promoting chicken myoblast proliferation [19,20]. Therefore, we concluded that miR-146b-3p inhibited chicken myoblast proliferation and differentiation and promoted apoptosis.

To well grasp the biological effects of miRNAs, their target genes are supposed to be taken into consideration. In this study, we found miR-146b-3p can target and inhibit the expression of *AKT1* and *MDFIC* and downregulate the activity of the *PI3K/AKT* pathway simultaneously.

*AKT1*, present in a variety of cell types, is widely distinguished as a key factor mediating numerous cellular activities including cell proliferation and differentiation [38,39,40]. In our present study, *AKT1* showed positive effects on myoblast proliferation and differentiation, coincidently contrary to those of miR-146b-3p. Besides, a variety of cell death paradigms have identified *AKT1* as a vital anti-apoptotic gene [41,42,43]. It is well established that *AKT1* is a vital gene in the *PI3K/AKT* pathway. An important function of activated PI3K in cells is the inhibition of programmed cell death, and AKT, a perfect candidate of PI3K, can serve in favor of *PI3K* to mediate cell-survival and cell-apoptosis responses [35,44,45]. Here, our results provide another piece of important evidence that *AKT1* is an essential suppresser of the myoblast apoptosis. Of note, the Western blot showed not only the protein abundance and the phosphorylation level of AKT1, but also the phosphorylation level of AKT that was dramatically suppressed by miR-146b-3p (Figure 4b,c), which can cause the downregulation of the *PI3K/AKT* pathway activity. The decline of the *PI3K/AKT* pathway activity caused by miR-146b-3p can lead to the inhibition of myoblast proliferation and differentiation and the promotion of myoblast apoptosis. Thus, our results collectively suggest that miR-146b-3p can suppress *PI3K/AKT* pathway by directly targeting *AKT1*.

When we were searching the targets for miR-146b-3p, the gene named *MDFIC* caught our eyes. As its name implies, MDFIC is an inhibitor of the MyoD family, which suppresses the myoblast differentiation. However, according to our preliminary reply verification, the overexpression of *MDFIC* can reverse the inhibitory effects of miR-146b-3p on chicken myoblast differentiation (Figure 4f), which aroused our interest to further detect how exactly *MDFIC* acts in the development of chicken myoblast. It was unexpected but our data did show that *MDFIC* promoted not only myoblast differentiation but also proliferation in chicken.

MyoD and MyoG, two essential members of the MyoD family, are upregulated to promote and maintain the process of myoblast differentiation [2,46]. Here, we are pleasantly surprised that *MDFIC* exhibited positive impacts on the mRNA expression of *MyoD* and *MyoG* in chicken myoblast (Figure 6f). Moreover, immunofluorescence also provided a convincing demonstration that *MDFIC* can promote myotubes formation in chicken (Figure 6i,j). The article that originally identified I-mfa as a myogenic repressor interacting with members of the MyoD family described their animal material was mouse and the transfected cells were NIH3T3 cells, a kind of fibroblasts [36]. While in our study, the animal material was chicken and the transfected cells were QM-7 cells and CPMs, both are myoblasts. Our experiment materials are completely different from those of the above study. The function of *MDFIC* can be different, depending on species and cell types. 

Here, we identified *MDFIC* as a novel factor promoting chicken myoblast proliferation and differentiation and inhibiting chicken myoblast apoptosis. As a matter of fact, to date, there are no reports about the role of *MDFIC* in myoblast development of chicken. Therefore, our study is the first to uncover the exact function of *MDFIC* in this field.

Collectively, our study sheds light on a mechanism by which miR-146b-3p inhibits myoblast proliferation and differentiation and promotes myoblast apoptosis via suppressing *PI3K/AKT* pathway activity and *MDFIC* expression. These discoveries reveal a novel model of miR-146b-3p on regulating skeletal muscle development.

## Figures and Tables

**Table 1:** Primers used for RT-PCR.

Gene Name	Primer Sequences (5′–3′)	Size (bp)	Annealing Temperature (°C)	Accession Number
*AKT1*	F: CACACGCTGACAGAAAACCG	128	60	NM_205055.1
	R: AACAACTCCCCTCCGTTAGC			
*MDFIC*	F: CAATGGCAGCAAGAAGA	126	52	XM_416018.5
	R: AGAACAATGTTACAGAGGGT			
*Cyclin B2*	F: CAGTAAAGGCTACGAAAG	133	58	NM_001004369.1
	R: ACATCCATAGGGACAGG			
*Cyclin D1*	F: CAGAAGTGCGAAGAGGAAGT	188	58	NM_205381.1
	R: CTGATGGAGTTGTCGGTGTA			
*Cyclin D2*	F: AACTTGCTCTACGACGACC	150	58	NM_204213
	R: TTCACAGACCTCCAACATC			
*PCNA*	F: GTGCTGGGACCTGGGTT	217	58	NM_204170.2
	R: CGTATCCGCATTGTCTTCT			
*p21*	F: GAAGAGTTGTCCACGATAAGC	247	58	NM_204170.2
	R: TTCCAGTCCTCCTCAGTCC			
*CDKN1B*	F: GCTGTGCTGGGCTGAA	207	58	NM_204256.2
	R: GGACGAAAGGATGTGGG			
*Caspase-3*	F: TGGCCCTCTTGAACTGAAAG	106	61	NM_204725.1
	R: TCCACTGTCTGCTTCAATACC			
*Caspase-8*	F: CCCTGAAGACAGTGCCATTT	207	61	NM_204592.2
	R: GGGTCGGCTGGTCATTTTAT			
*Caspase-9*	F: TCCCGGGCTGTTTCAACTT	270	61	XM_424580.5
	R: CCTCATCTTGCAGCTTGTGC			
*Fas*	F: TCCACCTGCTCCTCGTCATT	78	61	NM_001199487.1
	R: GTGCAGTGTGTGTGGGAACT			
*Cyt c*	F: TGTCCAGAAATGTTCCCAGTGC	138	61	NM_001079478.1
	R: CCTTTGTTCTTATTGGCATCTGTG			
*MYOD*	F: GCTACTACACGGAATCACCAAAT	200	58	NM_204214.2
	R: CTGGGCTCCACTGTCACTCA			
*MYOG*	F: CGGAGGCTGAAGAAGGTGAA	320	58	NM_204184.1
	R: CGGTCCTCTGCCTGGTCAT			
*MYHC*	F: CTCCTCACGCTTTGGTAA	213	58	NM_001319304.1
	R: TGATAGTCGTATGGGTTGGT			
*GAPDH*	F: TCCTCCACCTTTGATGCG	146	50–62	NM_204305.1
	R: GTGCCTGGCTCACTCCTT			

**Table 2:** Oligonucleotide sequences used in this study.

Sequence Name	Sequences (5′–3′)
gga-miR-146b-3p mimic	GGGAUACCUAAGUCAAGACG
gga-miR-146b-3p inhibitor	CGUCUUGACUUAGGUAUCCC
si-*AKT1*	GCTGAAGAAATGGAAGTTT
si-*MDFIC*	ATGGAAGTGGAATGCACAA

**Table 3:** Primers used for plasmids construction.

Primer Name	Primer Sequences (5′–3′)	Size (bp)	Annealing Temperature (°C)
pcDNA3.1-*AKT1*	F: CCC**AAGCTT**GAGACATTCCCGCCATTA	1612	61
	R: CCG**CTCGAG**AGAATCTGTGAGGCTTCCTA		
pcDNA3.1-*MDFIC*	F: CCC**AAGCTT**GTTAGTTCGGCTGGAGGGAG	1064	60
	R: CCG**CTCGAG**CAGCAGGCCGATAGTTCAGT		
pmirGLO-*AKT1*-3′UTR-WT	F: CCC**AAGCTT**AAAAAAATTAAAAAAGCC	100	55
	R: CCG**CTCGAG**CAAAAGGAAGGAGGAACC		
pmirGLO-*AKT1*-3′UTR-MT	F: AGAACTGAACATTCCCTAATAAGCGGGAGCAAA	2771	68
	R: TCTTGACTTGTAAGGGATTATTCGCCCTCGTTT		
pmirGLO-*MDFIC*-3′UTR-WT	F: CCC**AAGCTT**GGACTTTCTTTCTGTATTTAT	100	52
	R: TT**GCGGCCGC**AATTCTGCTTGTTTTAATTACA		
pmirGLO-*MDFIC*-3′UTR-MT	F: GTAATTTTCACTCCATAGGCAATCTTATAAGC	2771	68
	R: GCTTATAAGATTGAACGCTTAGTGAAAATTAC		

Bold sequence represents restriction enzyme recognition sequence.

## Supplementary Materials

The following are available online at https://www.mdpi.com/2073-4409/8/7/656/s1, Figure S1: The transfection efficiency of plasmids and RNA oligonucleotides. Figure S2: The relative mRNA expression levels of cell differentiation-related genes after transfection of miR-146-3p mimic or inhibitor in QM-7 cells. Figure S3: The effects of miR-146b-3p, *AKT1* and *MDFIC* on the protein levels of lamin B. Figure S4: The target relationship between miR-146b-3p and *AKT1* or *MDFIC*. Figure S5: The effects of *AKT1* or *MDFIC* on proliferation in QM-7 cells. Figure S6: The effects of *AKT1* or *MDFIC* on differentiation and apoptosis in QM-7 cells.
