# Supplementary figures and images for: The Inhibition on MDFIC and PI3K/AKT Pathway Caused by miR-146b-3p Triggers Suppression of Myoblast Proliferation and Differentiation and Promotion of Apoptosis
Source: Cells. 2019 Jun 29;8(7):656. doi: 10.3390/cells8070656 (PMC6678156; doi:10.3390/cells8070656)

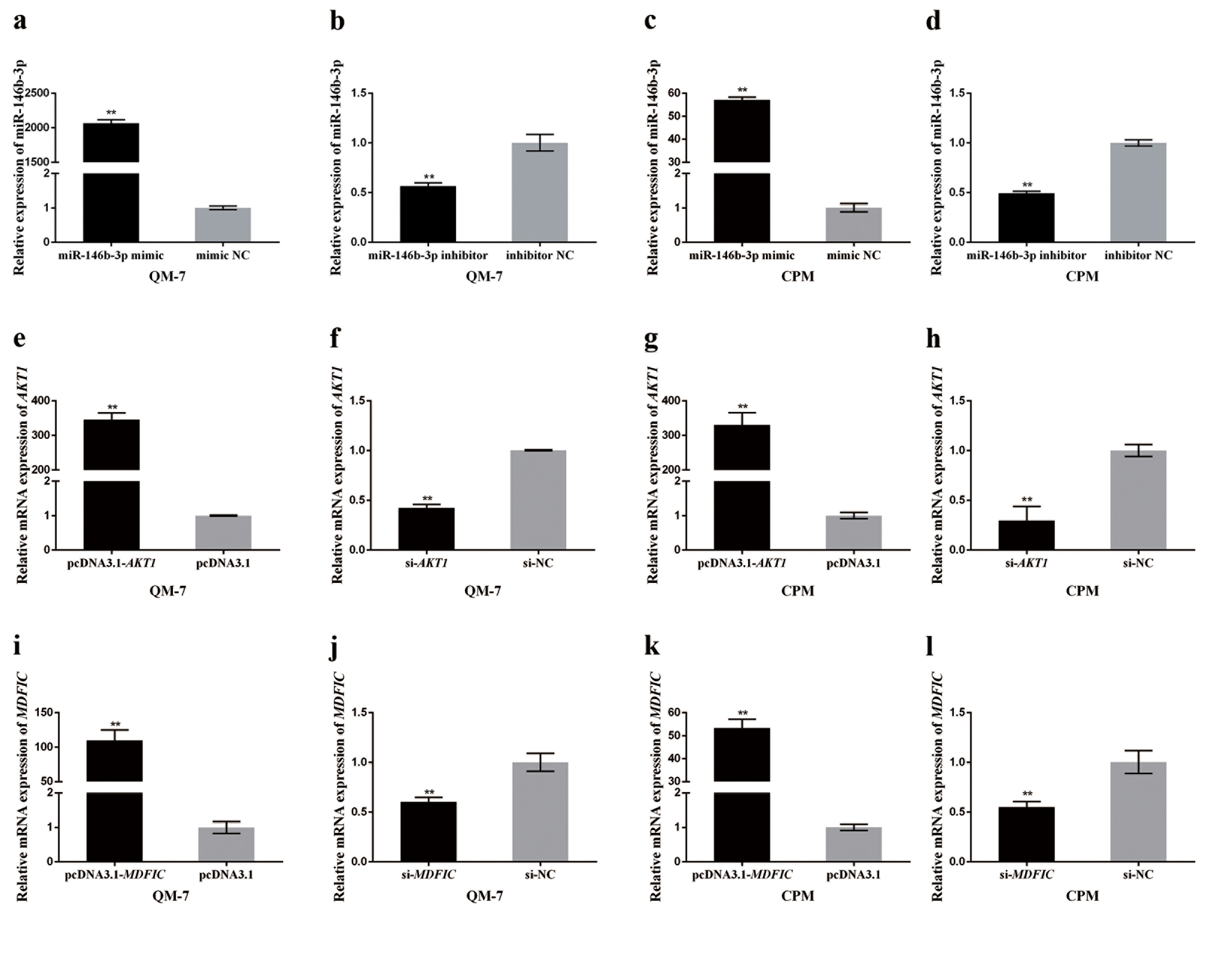

Supplement: Supplementary file 1 [file cells-08-00656-s001.zip › Supplementary Figure 1.jpg]

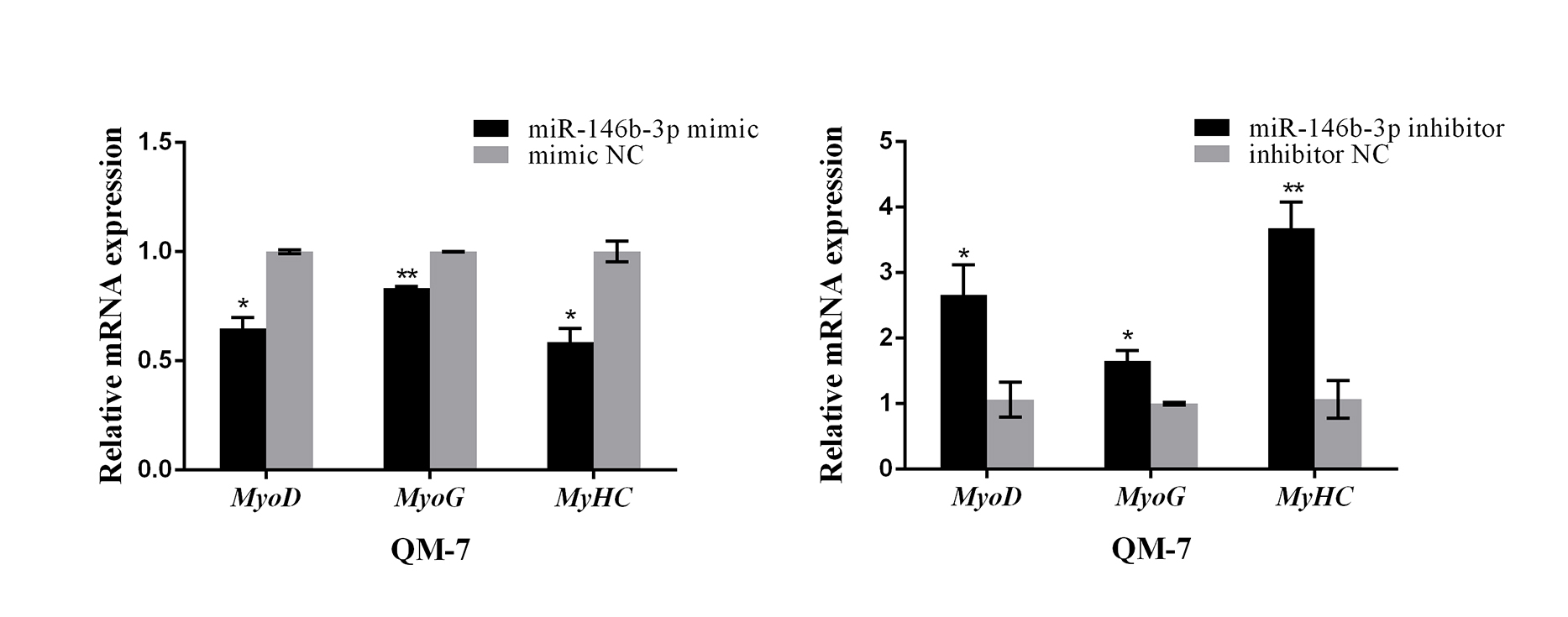

Supplement: Supplementary file 1 [file cells-08-00656-s001.zip › Supplementary Figure 2.jpg]

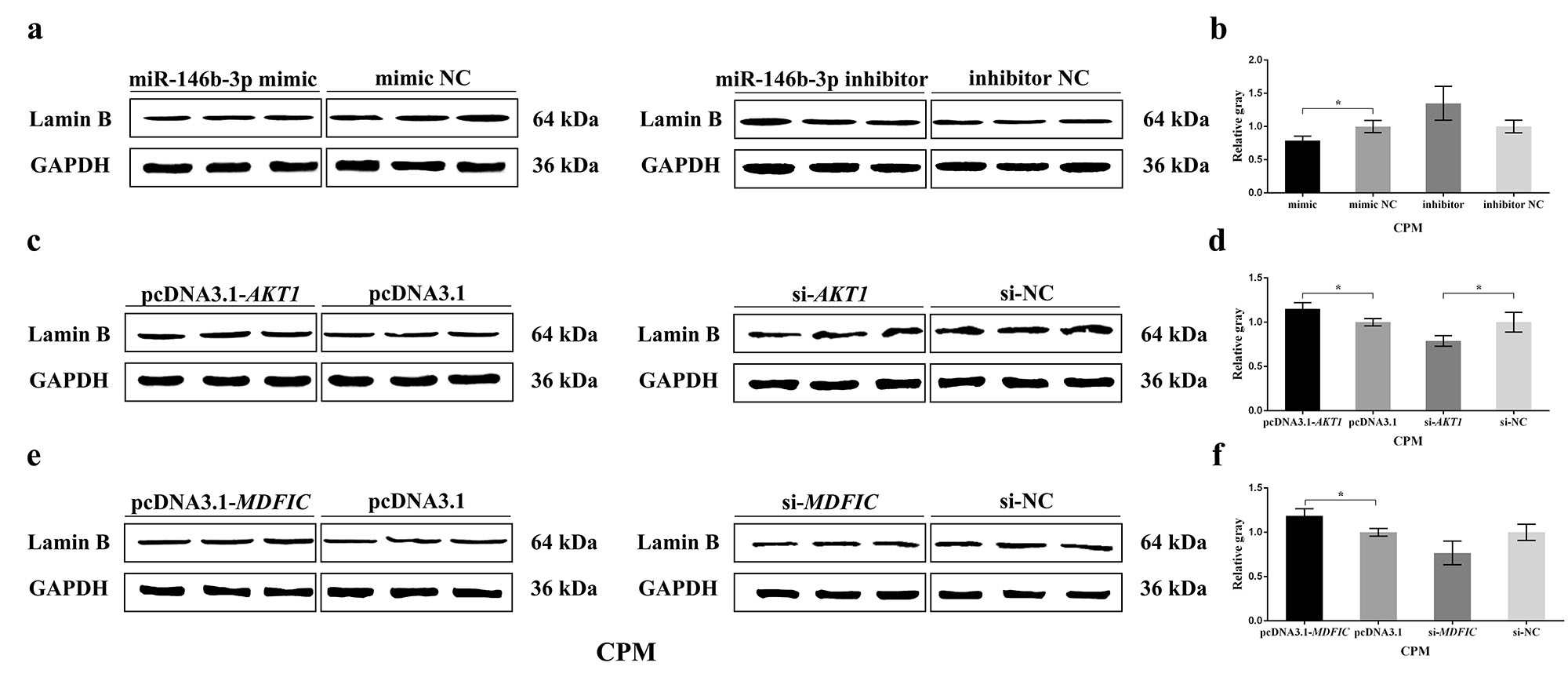

Supplement: Supplementary file 1 [file cells-08-00656-s001.zip › Supplementary Figure 3.jpg]

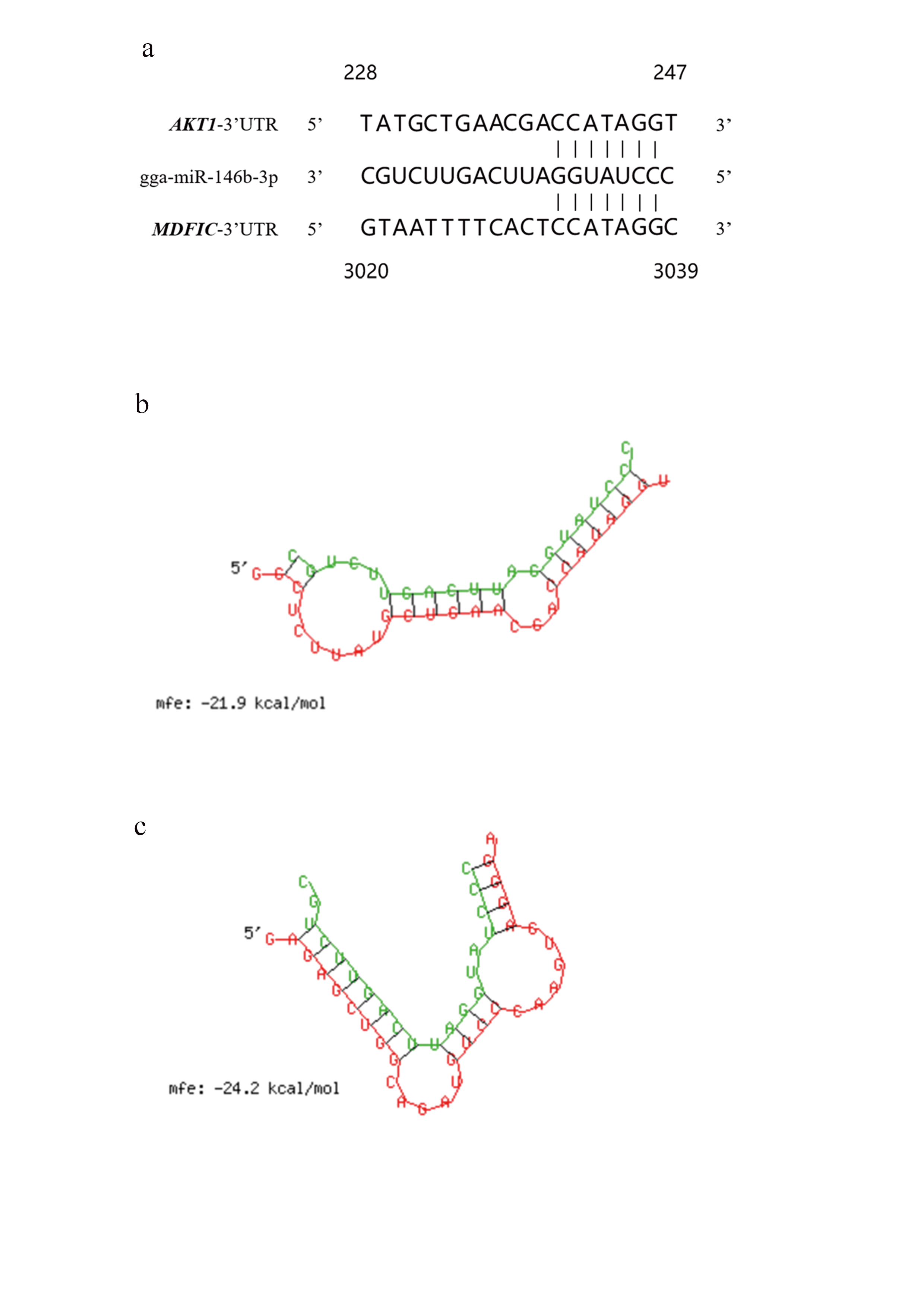

Supplement: Supplementary file 1 [file cells-08-00656-s001.zip › Supplementary Figure 4.jpg]

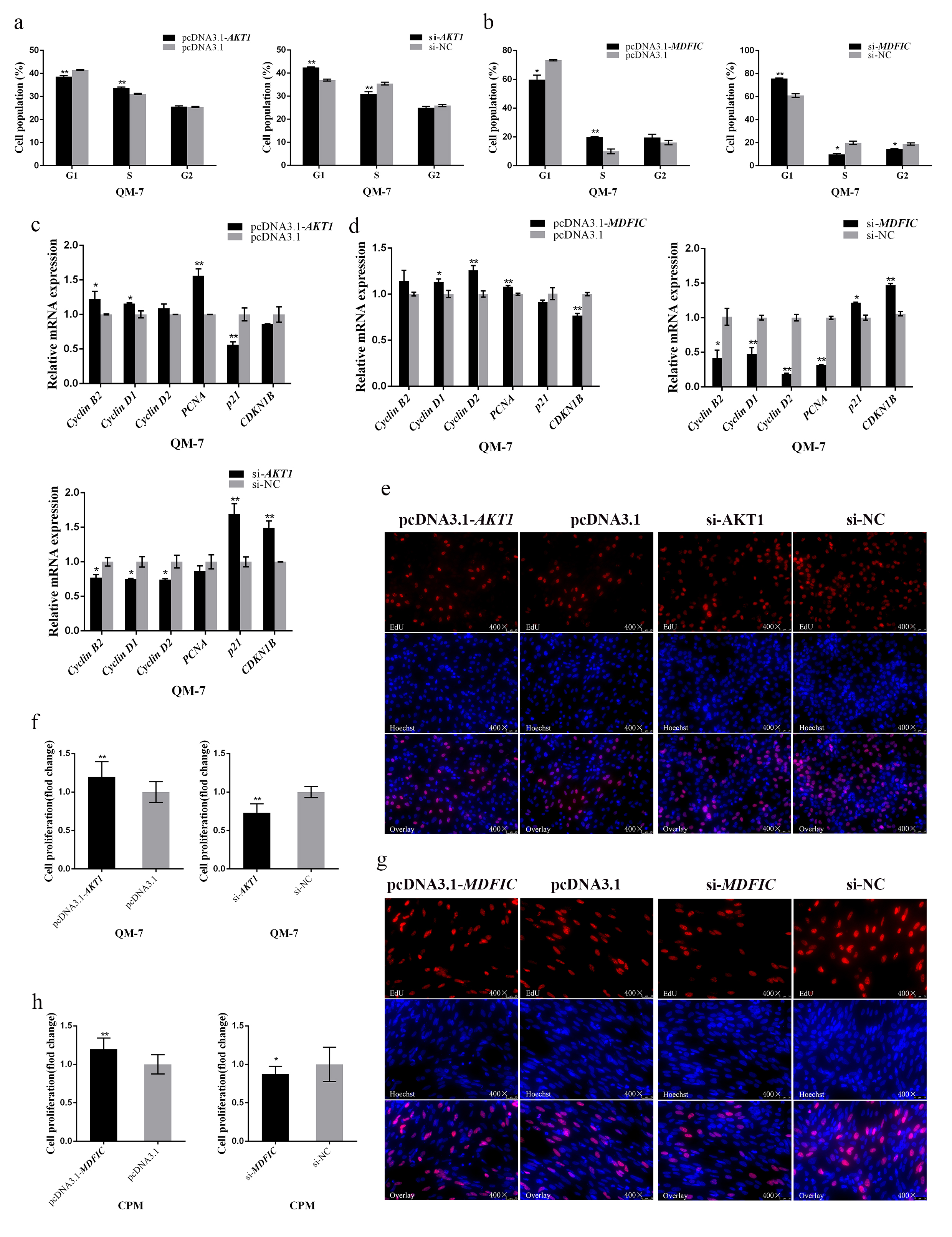

Supplement: Supplementary file 1 [file cells-08-00656-s001.zip › Supplementary Figure 5.jpg]

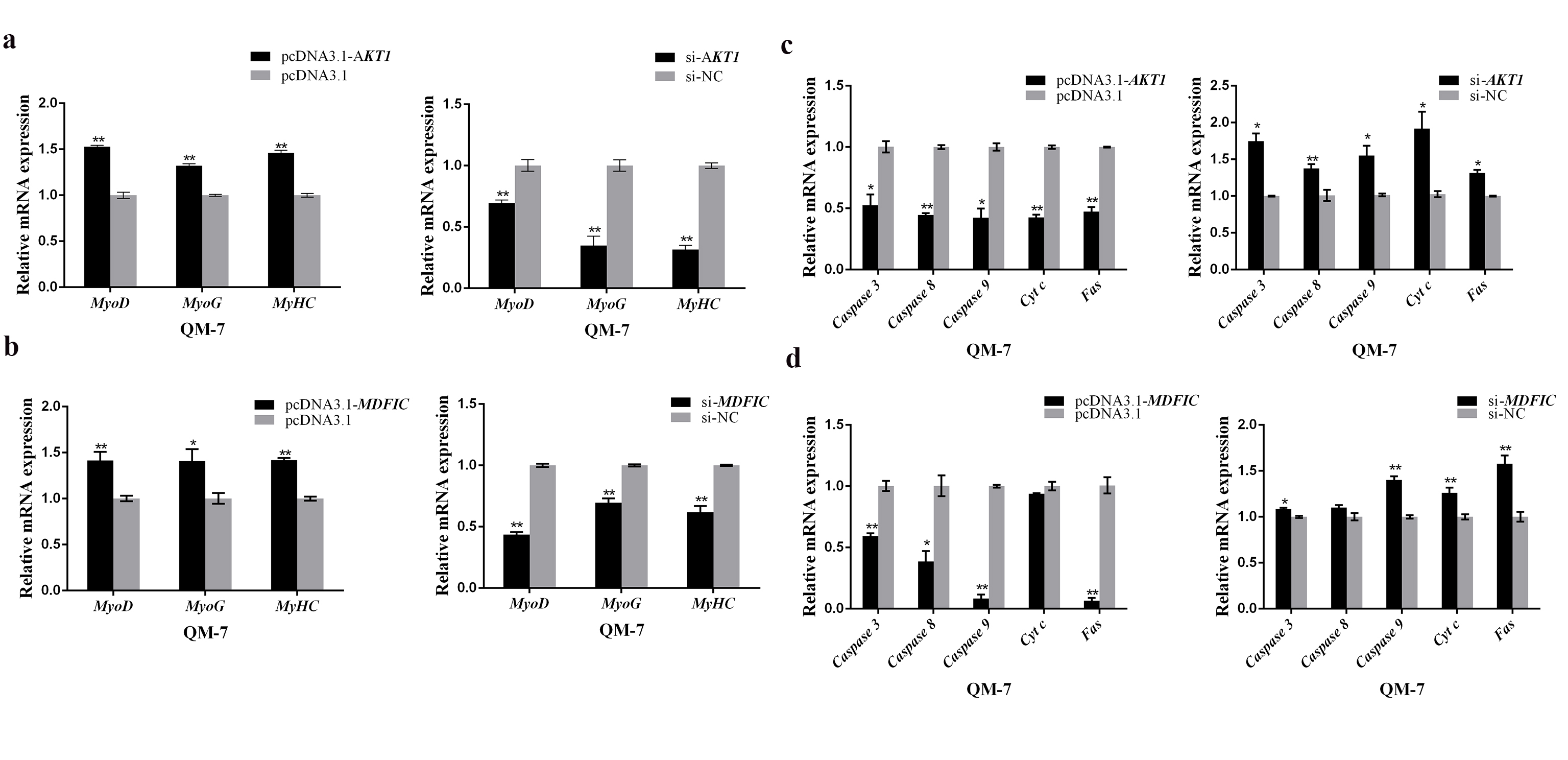

Supplement: Supplementary file 1 [file cells-08-00656-s001.zip › Supplementary Figure 6.jpg]
